# Antidepressant Treatment Normalizes White Matter Volume in Patients with Major Depression

**DOI:** 10.1371/journal.pone.0044248

**Published:** 2012-08-30

**Authors:** Ling-Li Zeng, Li Liu, Yadong Liu, Hui Shen, Yaming Li, Dewen Hu

**Affiliations:** 1 College of Mechatronics and Automation, National University of Defense Technology, Changsha, Hunan, People’s Republic of China; 2 Department of Psychiatry, First Affiliated Hospital, China Medical University, Shenyang, Liaoning, People’s Republic of China; 3 Department of Nuclear Medicine, First Affiliated Hospital, China Medical University, Shenyang, Liaoning, People’s Republic of China; Institute of Automation, Chinese Academy of Sciences, China

## Abstract

**Objective:**

To investigate white matter volume abnormalities in patients with major depression and the effects of antidepressant treatment on white matter volume.

**Method:**

Magnetic resonance imaging (MRI) was performed on 32 treatment-naïve depressed patients, 17 recovered patients who had received antidepressant treatment and subsequently achieved clinical recovery and 34 matched controls.

**Results:**

Relative to the healthy controls, the treatment-naïve depressed patients showed increased white matter volumes in the left dorsolateral prefrontal cortex (DLPFC) and left putamen and reduced white matter volumes in the left cerebellum posterior lobe and left inferior parietal lobule. For the treatment-naïve patients, the length in months of the current depressive episode was positively correlated with the white matter volumes in both the left DLPFC and left putamen. In the recovered patients, the differences in white matter volume were no longer statistically significant relative to healthy controls. No significant difference was found in the total white matter volume among the three groups.

**Conclusions:**

This study demonstrates that there were alterations in the white matter volumes of depressed patients, which might disrupt the neural circuits that are involved in emotional and cognitive function and thus contribute to the pathophysiology of depression. The finding of the significant correlations between refractoriness and the white matter volumes in the left DLPFC and left putamen combined with the finding that antidepressant treatment normalized the white matter volume of recovered patients, suggests that a quantitative, structural MRI measurement could act as a potential biomarker in depression therapy for individual subjects.

## Introduction

Major depressive disorder is a common mental disorder that is characterized by persistent, pervasive affective (e.g., depressed mood or anhedonia, a sense of worthlessness) and cognitive symptoms (e.g., working memory deficits, executive function impairments) [1]. Major depression has been ranked as a leading cause of living on disability worldwide and leads to serious social and economic problems [2]. To date, the pathophysiological mechanisms underlying affective and cognitive dysfunctions in depression remain unclear. Therefore, the search for neuroimaging-based biomarkers for the diagnosis and treatment of depression is an international imperative.

Major depressive symptoms are proposed to be associated with brain network dysregulation [3], [4]. Using resting-state functional magnetic resonance imaging (fMRI) techniques, a number of studies have detected network alterations in depressed patients, especially abnormalities in the default mode network [5] and cognitive control network [6]. Structural gray matter abnormalities might contribute to the functional abnormalities that are observed in depressed patients. In the past decade, structural MRI has been widely used to study brain alterations during depression, and major depression has been associated with structural brain abnormalities [3], [7], [8]. Because white matter fibers connect anatomically separated brain regions to form brain functional networks, major depression might also be associated with white matter alterations [9]. Using diffusion tensor imaging (DTI), microstructural changes in white matter during depression have been reported in the dorsolateral prefrontal cortex (DLPFC) [10], [11], cerebellum and hippocampus [12]. Structural MRI has also revealed white matter volume changes in depressed patients. Steingard et al. [13] and Bell-McGinty et al. [14] found significant frontal white matter reductions in depressed patients relative to healthy controls. Potter et al. reported greater white matter volumes in the left prefrontal cortices of patients with major depression [15]. However, several studies reported no differences between the white matter volume of depressed patients and healthy controls [12], [16]. These results are somewhat controversial and need to be clarified. To the best of our knowledge, few previous studies have examined the changes in white matter volume that occur in patients with major depression after successful antidepressant therapy.

In the current study, we used optimized voxel-based morphometry (VBM) combined with the New Segment procedure and the Diffeomorphic Anatomical Registration Through Exponentiated Lie algebra (DARTEL) toolbox [17] to investigate the extent to which white matter volume changes were present in treatment-naïve patients with major depressive disorder relative to healthy controls. In addition, it is not clear whether antidepressant therapy normalizes white matter volume in depressed patients. To address this issue, we conducted a structural MRI study of depressed patients before and after antidepressant treatment and then examined the effect of antidepressant treatment on white matter volume by comparing the white matter volumes of recovered patients who underwent treatment with antidepressant medications and subsequently achieved clinical recoveries with those of healthy controls. We predicted that recovered patients would no longer show a significant difference in white matter volume relative to the healthy group.

## Methods

### Participants

The depressed patients (N = 32, 10 males/22 females) were right-handed outpatients at the First Affiliated Hospital of China Medical University and were matched with 34 demographically similar healthy controls (N = 34, 8 males/26 females). All patients met the criteria for a current episode of unipolar recurrent major depression based on the DSM-IV criteria [1]. Using the Structured Clinical Interview for DSM-IV [18], confirmations of the patients’ diagnoses were made by clinical psychiatrists. The exclusion criteria included acute physical illness, substance abuse or dependence, history of a head injury resulting in loss of consciousness and a major psychiatric or neurological illness other than depression. The patients were abstinent from caffeine, nicotine and alcohol prior to scanning session. Patients had never been on medication for depression before their first scans and had scores on the 17-item Hamilton Depression Rating Scale (HDRS) [9] of 25.88±4.80 (18 to 38), Hamilton Anxiety Rating Scale (HAMA) [19] of 20.13±4.93 (8 to 30) and Clinical Global Impression Scale-Severity Scale (CGI-S) [20] of 5.81±0.64 (5 to 7). Control participants had HDRS and HAMA scores of 4.16±1.04 (2 to 6) and 3.44±0.93 (2 to 5), respectively. Before the first scans, the patients had experienced 1.97±1.33 previous episodes, and the current episode durations lasted an average of 7.78±9.13 months. Because some patients did not want to participate in the study a second time, 17 patients (3 males/14 females) were investigated again after successful serotonin and norepinephrine reuptake inhibitor (SNRI) or selective serotonin reuptake inhibitor (SSRI) treatment (following 12–16 weeks of treatment); these patients achieved clinical recoveries with HDRS scores ≤8 (5.18±1.24) and HAMA scores ≤6 (3.94±1.48) and remained well at >6 months of follow-up time. All participants provided written informed consent, and the present study was approved by the Ethics Committee of China Medical University.

### MRI Scanning

Magnetic resonance images were acquired using a 1.5-T GE SIGNA scanner (GE Medical Systems) with a volumetric 3D Flair Spoiled Gradient Recall (FSPGR) sequence. To reduce head movement, the subjects’ heads were fixed using foam pads with a standard birdcage head coil. For the analysis of white matter volumes, a high-resolution T1-weighted image was acquired with the following scan parameters: repletion time (TR) = 9.4 ms; echo time (TE) = 4.2 ms; flip angle = 15°; 164 sagittal slice; no slice gap; field of view = 260×260 mm; image matrix = 512×512; slice thickness = 1.0 mm.

### Data Preprocessing and Analysis

There were no artifacts or structural abnormalities in any of the structural MRI images by visual inspection. The images were preprocessed using SPM8 (Wellcome Department of Cognitive Neurology, Institute of Neurology, London, UK, http://www.fil.ion.uncl.ac.uk/spm). First, the New Segment procedure was performed to segment the structural MRI images into six partitions, including gray matter, white matter, cerebrospinal fluid and three other background partitions based on a modified mixed model cluster analysis technique. Then, a template was generated from the entire image dataset using the DARTEL procedure, and the resulting images were spatially normalized into the MNI space using an affine spatial normalization. Finally, the white matter images were spatially normalized to the relative template and smoothed with an 8-mm full width at half maximum isotropic Gaussian kernel.

Two sample two-tailed *t*-tests and Pearson Chi-square tests were used to compare the demographic data across the three participant groups. In addition to the paired *t*-test between the two scans of the recovered patients, we performed two-sample t-tests by using SPM8 to investigate the differences in the white matter volumes between other pairs of groups. Anatomical regions with significant white matter volume changes between pairs of groups were yielded based on a voxel-level height threshold of *p*<0.05 (FWE corrected) and a cluster size threshold of 20 voxels. Additionally, a Spearman's rank correlation analysis was performed to assess the correlations between the white matter volumes of the regions with group difference and clinical variables, i.e., HDRS score, HAMA score and length in months of the current depressive episode. Age was included as a confounding covariate. Two sample two-tailed levels of significance were set at *p*<0.05 and were uncorrected for multiple comparisons in the correlation analysis.

## Results

### Demographic Data

Treatment-naïve depressed patients and healthy controls were matched by gender (*p* = 0.482 with a Pearson’s Chi-square test), age (patients: 33.09±11.33 years vs. controls: 34.75±10.8 years, *p* = 0.709 with a two-tailed t-test) and education (patients: 11.13±3.17 years vs. controls: 11.06±3.31 years, *p* = 0.666 with a two-tailed t-test). Recovered patients (age: 32.65±11.74 years, education: 11.88±3.28 years) and healthy subjects were gender-, age- and education- matched with *p* = 0.305, 0.668, and 0.676, respectively.

### Group Difference in White Matter Volumes

**Figure 1:**
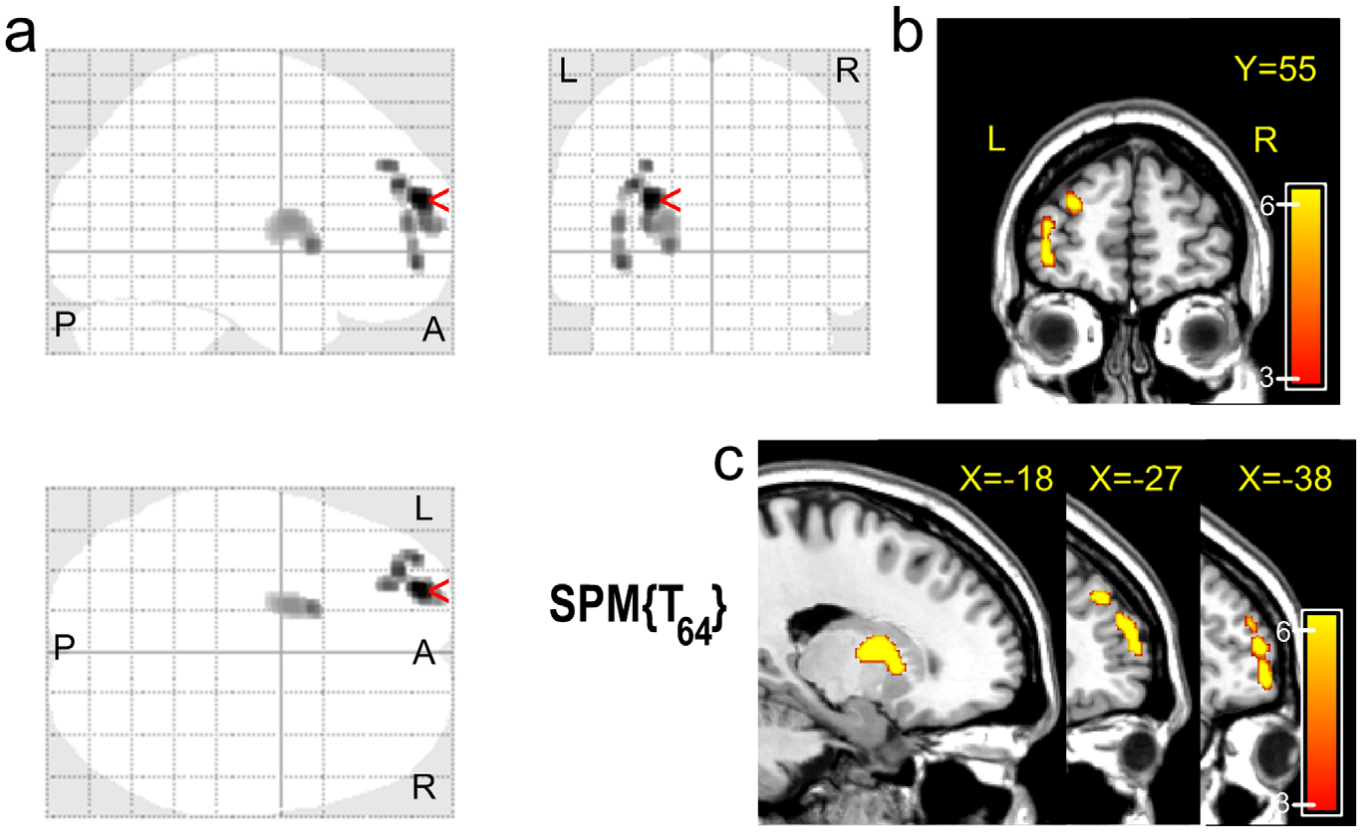
Anatomical regions with significant increases in white matter volume in treatment-naïve depressed patients relative to healthy subjects. Significance threshold with a family wise error (FWE) at *p*<0.05 and cluster size ≥20 voxels was used.

**Figure 2:**
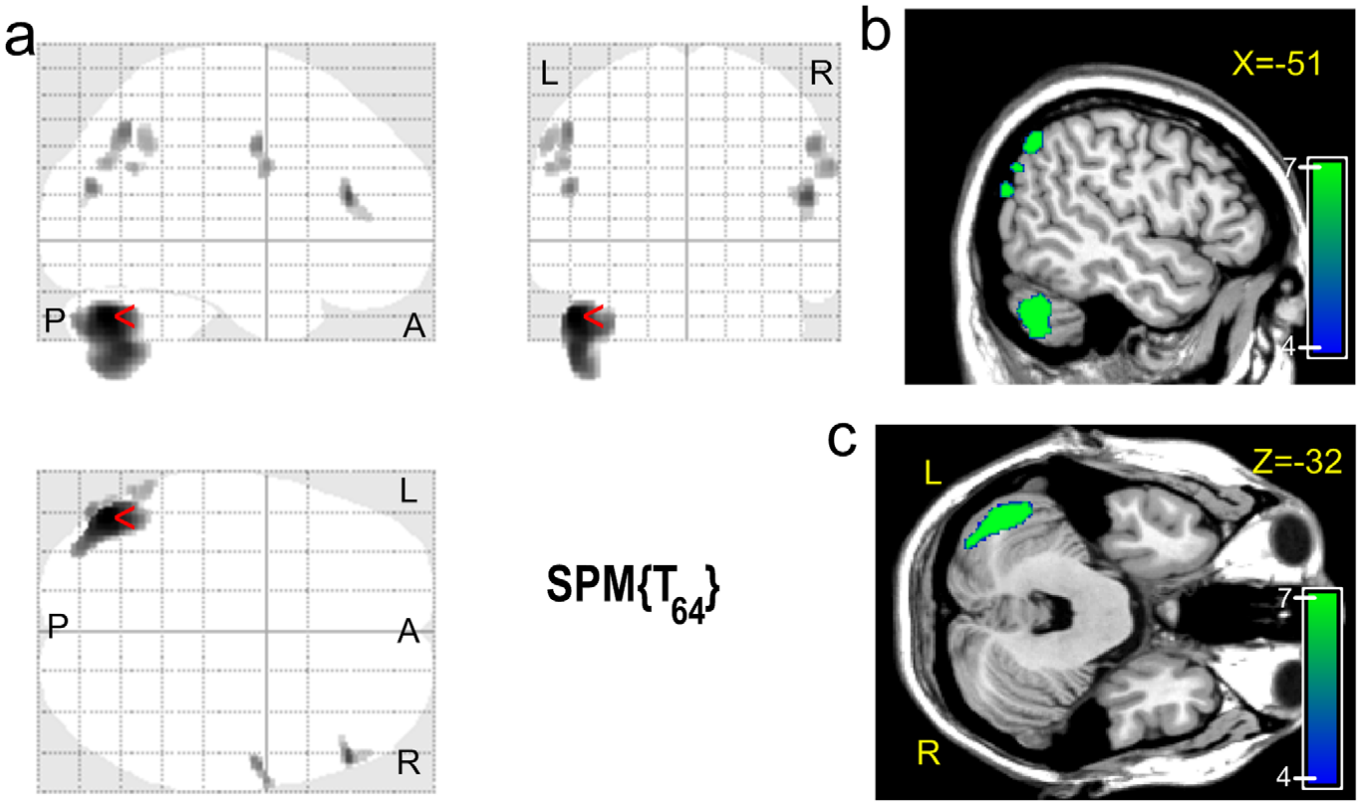
Anatomical regions with significant reductions in white matter volume in treatment-naïve depressed patients relative to healthy subjects. Significance threshold with a family wise error (FWE) at *p*<0.05 and cluster size ≥20 voxels was used.

**Figure 3:**
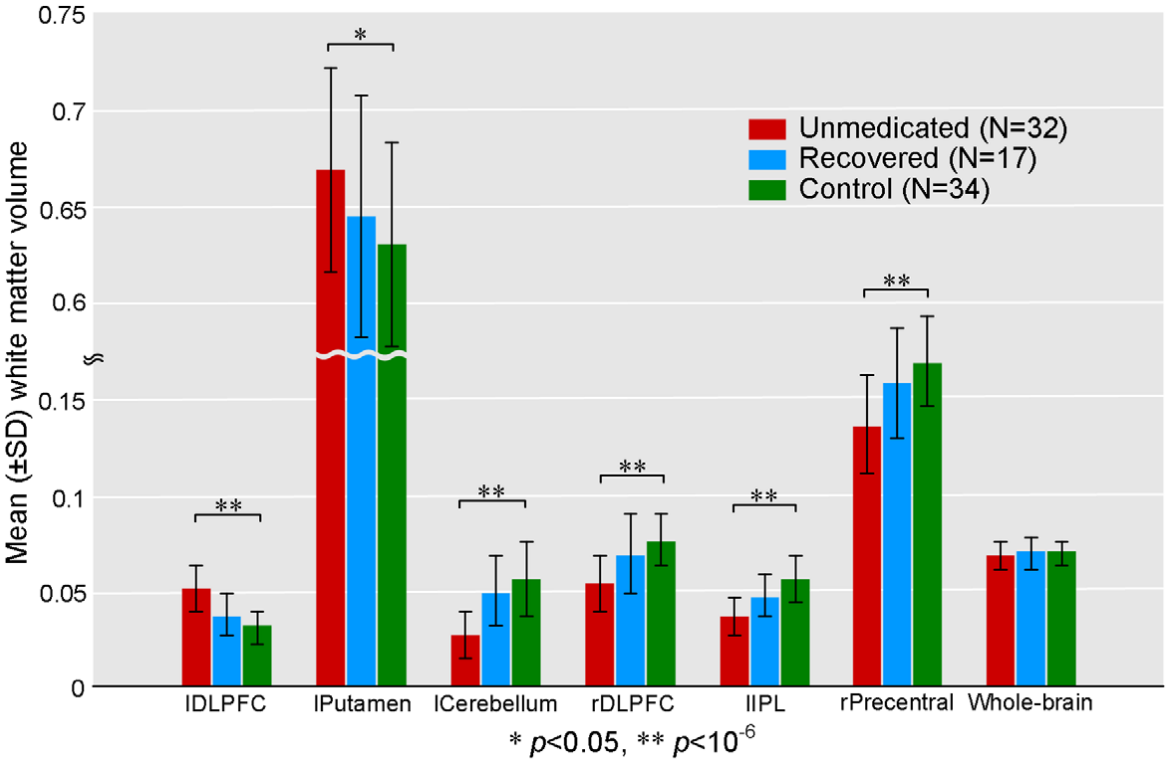
Voxel-averaged white matter volumes in the regions with significant changes in treatment-naïve depressed patients (N = 32), recovered patients (N = 17) and healthy subjects (N = 34). Two-tailed levels of significance (*p*<0.05, uncorrected) were used in the analyses. There were significant differences in the bilateral DLPFC, left putamen, left cerebellum posterior lobe, left inferior parietal lobule and right precentral gyrus between treatment-naïve depressed patients and healthy controls, but these differences did not remain statistically significant in the recovered patients after successful antidepressant therapy relative to the healthy group. There were no significant differences in the total white matter volume among the three groups. l/rDLPFC: left/right dorsolateral prefrontal cortex; lputamen: left putamen; lCerebellum: left cerebellum posterior lobe; lIPL: left inferior parietal lobule; rPrecentral: right precentral gyrus.

Relative to the healthy controls, treatment-naïve depressed patients showed significantly increased white matter volumes in the left DLPFC (BA 9/10) and left putamen and significantly decreased white matter volumes in the left cerebellum posterior lobe, right DLPFC (BA 46), left inferior parietal lobule (IPL, BA 39/40), left angular gyrus (BA 39) and right precentral gyrus (BA 6) (Table 1, Figures 1, 2, 3). However, these differences did not remain statistically significant in the recovered patients relative to healthy subjects (Figure 3). There was no significant difference in the total white matter volume among the three groups (Figure 3).

**Table 1:** Anatomical regions with significant white matter volume changes in treatment-naïve depressed patients relative to healthy subjects.

Anatomical regions	Side	BA	Cluster size	*t*-score	MNI coordinates (x, y, z)		
Increases							
Superior Prefrontal Gyrus	L	10	366	6.58	−25.5	58.5	21
	L	9	52	5.96	−27	45	36
Middle Prefrontal Gyrus	L	10	157	6.08	−37.5	57	−4.5
Putamen	L		409	5.91	−18	12	3
Reductions							
Cerebellum Posterior Lobe	L		1851	7.02	−48	−70.5	−31.5
Middle Frontal Gyrus	R	46	108	6.32	52.5	34.5	18
Inferior Parietal Lobule	L	39	146	6.04	−51	−63	46.5
	L	40	26	5.67	−58.5	−58.5	30
	L	40	60	5.65	−60	−52.5	42
Angular Gyrus	L	39	61	6.01	−49.5	−76.5	22.5
Precentral Gyrus	R	6	110	5.94	55.5	−4.5	40.5

*p*<0.05 (FWE corrected) with cluster size ≥20 voxels; L = left hemisphere; R = right hemisphere; BA = Brodmann area; MNI: Montreal Neurologic Institute.

To test the longitudinal changes of white matter volume, we performed paired t-tests using the two scans of the recovered patients. It was found that no voxels met the voxel-level height threshold of *p*<0.05 (FWE corrected) and a cluster size threshold of 20 voxels. Thus, a relaxed threshold of *p*<0.01 (FDR corrected) with cluster size >20 voxels was used here. After antidepressant treatment, the patients showed significantly reduced white matter volume mainly within the left putamen, left DLPFC (BA 9/10) and left precentral gyrus (BA 6), while showed significantly increased white matter volume primarily in the left IPL (BA 39/40), bilateral precentral gyri (BA 6), left orbital frontal cortex (BA 47), bilateral inferior and middle temporal gyri (BA 20/21/37), bilateral cerebellum posterior lobe and left lingual gyrus (BA 18) (Table 2).

**Table 2:** Anatomical regions with significant longitudinal changes of white matter volume in the recovered depressed patients.

Anatomical regions	Side	BA	Cluster size	*t*-score	MNI coordinates (x, y, z)		
Reductions							
Putamen	L		701	9.49	−24	−7.5	12
Middle Frontal Gyrus	L	10	1567	8.84	−25.5	52.5	13.5
	L	8	50	7.76	−37.5	15	49.5
	L	46	30	6.41	−45	37.5	18
Orbital Frontal Cortex	L	10	73	7.22	−7.5	67.5	−7.5
Superior Frontal Gyrus	L	9	76	6.89	−33	37.5	36
Precentral Gyrus	L	6	138	8.00	−31.5	−12	54
Increases							
Inferior Parietal Lobule	L	39/40	5412	8.01	−48	−55.5	40.5
Orbital Frontal Cortex	L	47	147	8.04	−33	31.5	−15
	R	47	2313	7.77	46.5	43.5	−4.5
Precentral Gyrus	R	6	1425	7.96	48	−10.5	34.5
	L	6	32	5.61	−52.5	−21	36
Inferior Temporal Gyrus	R	20/37	324	7.19	55.5	−33	−16.5
	R	20	93	6.66	55.5	−16.5	−31.5
	L	37	144	6.08	−48	−66	−6
	L	37	28	5.72	−57	−54	−10.5
Middle Temporal Gyrus	L	22	135	6.36	−54	−63	4.5
	L	21	20	5.67	−52.5	−28.5	−16.5
Lingual Gyrus	L	18	207	7.03	−10.5	−85.5	−6
	L	18	104	5.84	−21	−96	−10.5
Hippocampus	L		121	6.00	−18	−33	−4.5
Cerebellum Posterior Lobe	R		46	5.73	21	−67.5	−25.5
	R		314	6.46	46.5	−60	−42
	L		270	6.21	−34.5	−76.5	−30
	L		36	5.48	−51	−66	−30

*p*<0.01 (FDR corrected) with cluster size ≥20 voxels; L = left hemisphere; R = right hemisphere; BA = Brodmann area; MNI: Montreal Neurologic Institute.

### Clinical Correlation Analysis

**Figure 4:**
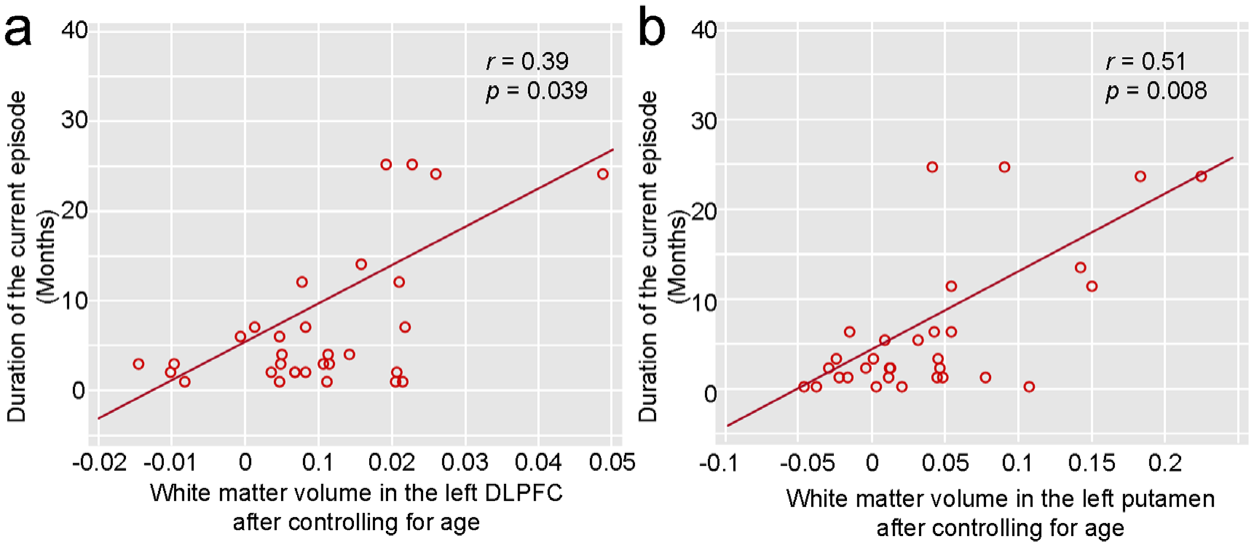
The white matter volumes in the left DLPFC cluster (a) and left putamen cluster (b) were significantly positively correlated with the duration of the current depressive episode among 32 treatment-naïve depressed patients when the outliers were excluded from analyses. DLPFC: dorsolateral prefrontal cortex.

Depression refractoriness, as measured by the length in months of the current depressive episode, was positively correlated with the white matter volumes in the left DLPFC cluster and left putamen cluster in treatment-naïve depressed patients, and the correlation coefficients were 0.35 (Fisher *r*-to-*z*, *p* = 0.057) and 0.48 (Fisher *r*-to-*z*, *p* = 0.011), respectively. If the point corresponding to the patient with longest duration of current depressive episode was treated as ‘outlier’ and was removed, the correlations remained significant (0.39, *p* = 0.039; 0.51, *p* = 0.008) (Figure 4). No regions were significant correlated with the HDRS or HAMA scores.

## Discussion

This study investigated the alterations in white matter volume that occurred in depressed patients and examined the effects of antidepressant therapy on white matter volume. Relative to the healthy controls, the treatment-naïve depressed patients showed significant white matter volume increases in the left DLPFC and left putamen and significant white matter volume reductions in the left cerebellum posterior lobe, right DLPFC, left IPL and angular gyrus and right precentral gyrus. Furthermore, in the treatment-naïve depressed patients, the length in months of the current depressive episode was positively correlated with the white matter volumes in the left DLPFC and left putamen. After antidepressant therapy, there was no significant difference in the white matter volumes of recovered patients relative to healthy subjects. No significant difference was found in the total white matter volume among the three groups.

The DLPFC, as a critical region in the cognitive control network [6], was altered with respect to the white matter volume in the depressed patients in the present study. The cognitive control network, which is involved in cognitive control and working memory function [21], is impaired in depression [6], [22]. Several recent fMRI studies have reported abnormal connectivity of the DLPFC in depressed patients [23], [24]. Recent DTI studies found abnormalities in the DLPFC white matter [10], [11]. The finding of white matter increase in the left DLPFC here is counter-intuitive, but it is not implausible as recent MRI studies also demonstrated the white matter increase in the left prefrontal cortex of depressed patients relative to healthy controls [15], [25]. In this study, white matter volume reduction was also observed in the right DLPFC, which may consist with the previous studies reporting decreased gray matter density in this region to some extent [26], [27]. We speculate that the white matter volume alteration in the DLPFC might disrupt the cognitive control network and thus contribute to the pathophysiology of major depression. On the other hand, after treatment with antidepressant medication, the patients showed the white matter volume reduction in the left DLPFC, and the difference in the white matter volume in the DLPFC was not statistically significant between the recovered patients and healthy controls. This result, to some extent, was consistent with previous studies reporting normalized glucose metabolism in the DLPFC after antidepressant therapy [28]. The within-group correlation analysis, demonstrating increased left DLPFC white matter volume with increasing length in months of the current depressive episode, suggested that the white matter volume of the left DLPFC might be a potential biomarker for refractoriness to treatment.

A larger white matter volume in the left putamen was found in treatment-naïve depressed patients relative to healthy controls. White matter abnormalities within the putamen might disrupt the functioning of the frontostriatal circuits, which might explain the anhedonia and reduction in goal-seeking behavior that is observed in depressed patients [29]. In treatment-naïve depressed patients, the length in months of the current episode was positively correlated with the white matter volume in the left putamen. Additionally, the white matter volume in the left putamen was normalized after antidepressant treatment. Though the biological basis of white matter volume increase in the left putamen remains unclear, these findings suggest that the white matter volume in the left putamen might serve as a potential biomarker in the clinical diagnosis and treatment of major depression.

In this study, a smaller white matter volume in the cerebellum posterior lobe was observed in treatment-naïve depressed patients, and this alteration was no longer statistically significant in recovered patients relative to healthy subjects. Many previous studies have demonstrated that the cerebellum posterior lobe is associated with higher order functions, including executive control, salience detection and episodic memory/self-reflection; therefore, the cerebellum posterior lobe might contribute to the cognitive control network [30], [31], [32]. Some fMRI studies have reported functional abnormalities in the left cerebellum posterior lobe in major depression [24], [33], [34], [35], [36]. A recent meta-analytic study has demonstrated reduced activation of cerebellum posterior lobe to the positive emotion in depressed patients relative to the healthy controls [37]. Besides, gray matter density reduction was also observed in the cerebellum in the previous studies [26], [27]. White matter volume reductions in the cerebellum are not commonly reported in major depression. We speculate that the smaller white matter volume in the cerebellum posterior lobe might contribute to functional abnormalities such as cognitive control deficits that are observed in depression. It should be noted that the white matter volume abnormalities in the cerebellum exhibited hemispheric lateralization in major depression in this study that may be in accordance with previous studies demonstrating structural or functional asymmetries in the cerebellum in healthy subjects and psychiatric patients [38].

The IPL together with the angular gyrus (mainly including BA 39), a core region in the default mode network [39] and the cortical-limbic circuits in depression [4], is thought to play a role in emotional modulation [40], [41]. Enhanced activation in this region has been found in response to stimuli consisting of sad words in depressed patients relative to controls [42]. A recent classification study has demonstrated white matter volume reduction in the left IPL in the patients with non-refractory depressive disorder relative to healthy controls [25]. The findings of functional imaging studies, coupled with the observed white matter volume loss in this area in depressed patients, might provide a neural basis for the impaired processing of complex emotional stimuli in major depression. In addition, the white matter volume was reduced in the right precentral gyrus in treatment-naïve depressed patients and was normalized in recovered patients after successful antidepressant therapy. These alterations might also reflect, to some extent, the functional abnormalities observed in these regions in depressed patients [43], [44].

It seems that the brain regions with longitudinal changes of white matter volume covered more areas relative to the regions with group differences between the treatment-naïve patients and healthy controls. In fact, if we relaxed the threshold and cluster size criterion in the analyses of white matter volume between the treatment-naïve patients and healthy controls, we would found that the two analysis results were keeping very well with each other. Besides, it seems that the outliers negatively influence the clinical correlation analysis to some extent. If the couple ‘outlier’ points were removed, the correlations between depression refractoriness and white matter volume in the given regions were enhanced in this study.

There are several limitations related to sample size, scanner variability and the lack of a large independent dataset with which to confirm the findings. Therefore, it is important to confirm the results with a larger sample size and multicenter imaging data. Moreover, all 32 depressed patients were found to be responsive to treatment with antidepressant therapy and to subsequently achieve clinical recoveries following 12–16 weeks of treatment; therefore, white matter volume alterations in different therapeutic responses of major depressive disorder, such as treatment-responsive and treatment-resistant depression, need to be investigated.

In summary, the white matter volume was increased in the left DLPFC and left putamen and was reduced in the left cerebellum posterior lobe, right DLPFC, left IPL, angular gyrus and right precentral gyrus in patients with major depression. After successful antidepressant therapy, the white matter volumes were not significantly different in the recovered patients relative to healthy controls. Our findings support the idea that the neural circuits that are involved in emotional and cognitive function might be disrupted by white matter alterations; thus, these alterations may contribute to the pathophysiology of major depression. Furthermore, the finding of correlations between refractoriness and white matter volumes in the left DLPFC and left putamen suggests that a quantitative, structural MRI measurement could serve as a potential biomarker in major depression therapy for individual subjects.
